# Prevalence of Klebsiella pneumoniae strains producing carbapenemases and increase of resistance to colistin in an Italian teaching hospital from January 2012 To December 2014

**DOI:** 10.1186/s12879-015-0996-7

**Published:** 2015-06-27

**Authors:** Saverio Giuseppe Parisi, Andrea Bartolini, Erica Santacatterina, Elena Castellani, Roberto Ghirardo, Alessandro Berto, Elisa Franchin, Nicola Menegotto, Ettore De Canale, Tiziana Tommasini, Roberto Rinaldi, Monica Basso, Stefania Stefani, Giorgio Palù

**Affiliations:** Department of Molecular Medicine, University of Padova, Via Gabelli 63, 35100 Padova, Italy; Microbiology and Virology Unit, Padova Hospital, Padova, Italy; Infectious Diseases Unit, Padova Hospital, Padova, Italy; Department of Biomedical and Biotecnological Sciences, University of Catania, Catania, Italy

**Keywords:** Klebsiella pneumoniae carbapenemase producing, Active surveillance, Rectal swabs, Colistin resistance, Multilocus sequence typing

## Abstract

**Background:**

The aim of this study was to characterize the spread of carbapenemase-producing *Klebsiella pneumoniae* (CPKP) in a tertiary level hospital using ongoing active surveillance with rectal swab cultures. Furthermore, this study analyzed the presence of CPKP in the clinical samples (CS) of a single patient as well as the evolution of Colistin-sensitive strains (CoS) to Colistin-resistant strains (CoR).

**Methods:**

This study was performed from January 1, 2012 to December 31, 2014. In 2012, a survey was conducted in the Intensive Care Department. In autumn 2013, active monitoring was extended to the Surgery Department, and since mid-2014, the surveillance has included the Medical Department as well. Only the first isolated strain from each patient was included. Antimicrobial susceptibility testing was performed on CPKP isolates: *Klebsiella pneumoniae* carbapenemase, oxacillinase-48, Verona integron-encoded metallo-β-lactamase and New Delhi metallo-β-lactamase were detected using a validated in-house PCR method, and multilocus sequence typing (MLST) was used to investigate the clonal transmission of strains.

**Results:**

A total of 15,104 patients were included in the study, and 496 consecutive non-replicated strains of CPKP were collected: 149 strains were collected in 2012 (39 [26.2 %] from surveillance rectal swabs [SRS]), 133 strains were collected in 2013 (70 [52.6 %] from SRS) and 214 strains were collected in 2014 (164 [76.6 %] from SRS). We observed a significant increase in the percentage of positive SRS cases in 2014 relative to 2013 and 2012 (*p* = 0.0001 and *p* = 0.0172, respectively) and in the proportion of CPKP first isolated by SRS relative to those identified by CS (*p* < 0.0001). Among all available samples, the number of CoR isolated from SRS was higher in 2013 and 2014 compared with 2012 (*p* = 0.0019 and *p* = 0.008, respectively). ST-258 and ST-512 were more prevalent in the tested specimens, and a new single locus variant (SLV) of ST-512 (ST-745) was isolated.

**Conclusions:**

The results of this 3-year study of 15,104 patients highlight the clinical relevance of antimicrobial resistance as well as the drug-selection pressure of colistin therapy. The active surveillance in the three different departments increased the level of CPKP cases isolated by SRS.

## Background

*Klebsiella pneumoniae* is a gram-negative that is associated with nosocomial and community-acquired infections. Strains of carbapenemase-producing *Klebsiella pneumoniae* (CPKP) have been identified since the 1990s and have rapidly spread in many countries [1]. The most widespread carbapenemase enzymes include class A carbapenemases (KPC types), class B or metallo-β-lactamases (Verona integron-encoded metallo-β-lactamase [VIM] and New Delhi metallo-β-lactamase types [NDM]) and class D oxacillinases (OXA-48-like enzymes) [2]. In addition, the decreased susceptibility of *Enterobacteriaceae* to carbapenems may be caused by either extended-spectrum beta-lactamases (ESBLs) or AmpC enzymes as well as decreased drug permeability caused by porin loss [3].

Giani et al. [4] described the first Italian report of CPKP in 2008; since then, its detection has increased steadily. A recent survey involving 21 Italian laboratories reported that most carbapenem-resistant *Enterobacteriaceae* isolated from clinical specimens between November 2013 and April 2014 were CPKP (93 %) [5]. The infections caused by these organisms are difficult to treat, and relatively few treatment options are available. The discovery and development of newer, effective antibiotics has declined, and old agents (e.g., colistin, particularly in combination with carbapenems) have become the therapy of last resort [6, 7]. Colistin (polymyxin E) is a bactericidal agent. Colistin sulfate and colistimethate sodium, a prodrug that is hydrolyzed to colistin sulfate, are the commercially available formats. Colistimethate sodium is administered parenterally and exerts its effect by interacting with lipopolysaccharide (LPS) molecules, leading to outer membrane failure and disruption [6]. Increased use of colistin for multidrug resistant *Enterobacteriaceae* infections has led to the emergence of colistin resistance among CPKP [5]; Monaco et al. [8] reported an overall resistance rate of 43 %. Even more worrisome, resistant strains were isolated at all included centers. Moreover, hospital outbreaks have been described in Greece (involving two hospitals) and in the United States (involving three different institutions) [9, 10]. The resistance mechanisms have been investigated, and the latest genomic analyses suggest that insertional inactivation of the mgrB gene, upregulation of the PhoP/PhoQ signaling system, activation of the PmrA-regulated pmrHFIJKLM operon and the presence of ArnB can lead to LPS alterations that are linked to colistin resistance in pathogens such as K. pneumoniae [6, 11, 12].

The acquisition of carbapenemase-producing organisms in the healthcare setting has serious implications for both single patient outcomes as well as for public health. Rectal surveillance cultures on admission allow for the timely detection of infected patients and for the application of infection control measures to contain KPC-producing organisms and to select an appropriate therapeutical choice [13, 14].

The Padua Teaching Hospital is a highly accessed tertiary care hospital with 1400 recovery beds and approximately 200 admissions per day. Since 2010, an increased incidence of CPKP clinical isolates has been reported; for this reason, rectal swabs are used for active surveillance of asymptomatic carriers.

The aim of this study was to characterize the spread of CPKP in a tertiary care setting using ongoing active surveillance, with a particular focus on the evolution of colistin-sensitive strains (CoS) into colistin-resistant strains (CoR) in a single patient.

## Methods

### Study design

This study was performed from January 1, 2012 to December 31, 2014. In 2012, a survey was conducted in the Intensive Care Department (ICD) upon admission and at least weekly thereafter. Subsequently, in autumn 2013, active monitoring was extended to all patients admitted to the Surgery Department (SD), and since mid-2014, the surveillance has included patients in the Medicine Department (MD) who were hospitalized in the last two months or who arrived from long-term care facilities. Some isolates were obtained during incidental surveys of subjects hospitalized in the same room or in the same ward of a positive subject. The study included both all clinical samples (CS) from adult hospitalized patients from whom CPKP was isolated and the rectal surveillance cultures. Only the first CPKP strain isolated from each patient was included. The overall prevalence was evaluated for each single year, by material (CS versus rectal swab) and by the susceptibility or resistance to colistin.

The study was approved by the Ethical Committee for Clinical Experimentation, Padua Province (Ethics Review 3418/AO/15). Patient consent to the study was not required as all samples were collected as part of routine management/surveillance, and were anonymised prior to research use. Throughout this period, infection control measures were strengthened, especially in the wards where a KPC was detected. In particular, isolation rooms for colonized/infected patients were set up, or the subjects were transferred to the infectious diseases ward. All contact precautions were improved. Isolation was not feasible in all cases because of insufficient bed capacity cases, but the colonized or infected patients were cohorted in the same room whenever it was possible.

### Phenotypic assays

A screening test for carbapenemase-production in rectal swabs was performed by inoculation on MacConkey II Agar (Becton, Dickinson and Company, MD, USA), with the automated WASP® system (Copan, Brescia, Italy). In addition, an ertapenem disk (10 μg, BD BBL™ Sensi-Disk™) was placed on the media to identify suspected carbapenemase-producing colonies (screening cut-off ≤ 25 mm) [15]. An ertapenem disk was used because of its high sensitivity in detecting carbapenemase-producing strains.

Clinical samples were treated with routinary methods, by using BD Chocolate Agar (GC II Agar with IsoVitaleX) and/or BD Columbia Agar with 5 % Sheep Blood and/or CHROMagar Orientation Medium (for urine samples).

Microbial identification was performed in all strains by using bioMérieux Vitek® 2 and Vitek® MS. Antimicrobial susceptibly testing was performed with a Vitek® 2 automated system. Strains that exhibited reduced susceptibility to carbapenems (an MIC value ≥ 1 mg/L for ertapenem and/or imipenem and/or meropenem) were also tested using the dilution method (Thermo Scientific Sensititre™ system) for confirmation. Other phenotypic methods employed for the detection and confirmation of carbapenemase were the modified Hodge test and the Rosco Diagnostica KPC/MBL confirmation kit. Colistin susceptibility, initially evaluated using the Vitek® 2 automated system, was then confirmed with the dilution method on all strains and with Etest® (on 52 strains in 2014) because of possible over-estimation of resistance by automated methods [16].

All MIC values were evaluated with EUCAST Clinical Breakpoint tables (v 4.0) [17].

### Genotypic assays

A validated in-house PCR method was used to detect KPC, KPC type, OXA-48, VIM, and NDM carbapenemases in cases of suspected carbapenemase-producing strains [18].

Multilocus sequence typing (MLST) was used according to the MLST website [19] for further characterization of the strains to investigate possible cases of intra-hospital transmission and not for research purposes.

### Statistical methods

Data were expressed as absolute numbers and percentages.

The Mann–Whitney *U* test was used to compare the median values between groups. The Chi-squared test and Fisher’s exact test were used to compare proportions (as appropriate), and the Chi-squared test for trends was used to evaluate the trends in proportions. Values of *p* < 0.05 were considered statistically significant.

All statistical analyses were performed with MedCalc Statistical Software version 14.12.0 (MedCalc Software bvba, Ostend, Belgium; http://www.medcalc.org; 2014).

## Results

Overall, 15,104 patients were included in the study (2,645 subjects in 2012, 5,249 in 2013 and 7,210 in 2014), and a total of 496 consecutive non-replicated strains of CPKP were collected, as first isolate detection from each patient.

The identification was performed with the Hodge test and confirmed in 496/496 strains. Rosco Diagnostica test was performed in a subset of 93/496 KPC/MBL isolates and we obtained the following results : in 2012, 23 strains showed synergism with meropenem and boronic acid, and all but one were tested and confirmed by molecular analysis; in 2013, 64 strains showed synergism with meropenem and boronic acid (all but 12 tested and confirmed by molecular analysis); 3 strains didn’t showed synergism and were confirmed as OXA-48; in 2014, 3 strains showed synergism with meropenem and boronic acid and were tested and confirmed by molecular tests. Four hundred thirty-six strains were characterized using molecular methods: 432 KPC strains, 3 OXA-48 strains and 1 NDM strain were identified.

**Fig. 1:**
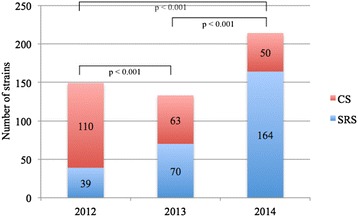
Carbapenemase producing *Klebsiella pneumoniae* strains isolated in 2012, 2013 and 2014. CS: clinical samples. SRS: surveillance rectal swabs

Specifically, we collected 149 CPKP strains in 2012 (39 [26.2 %] from screening rectal swabs [SRS]), 133 strains in 2013 (70 [52.6 %] from SRS) and 214 strains in 2014 (164 [76.6 %] from SRS). The number of CPKP strains isolated using SRS increased in 2013 relative to 2012 (*p* < 0.001) and in 2014 relative to 2012 and 2013; this increase was statistically significant (*p* < 0.001 and *p* < 0.001) (Fig. 1). A higher absolute percentage (relative to the number of patients) was reported in 2014 (2.3 %), and this difference was significant relative to 2013 (1.3 %, *p* = 0.0001) and 2012 (1.5 %, *p* = 0.0172).

**Fig. 2:**
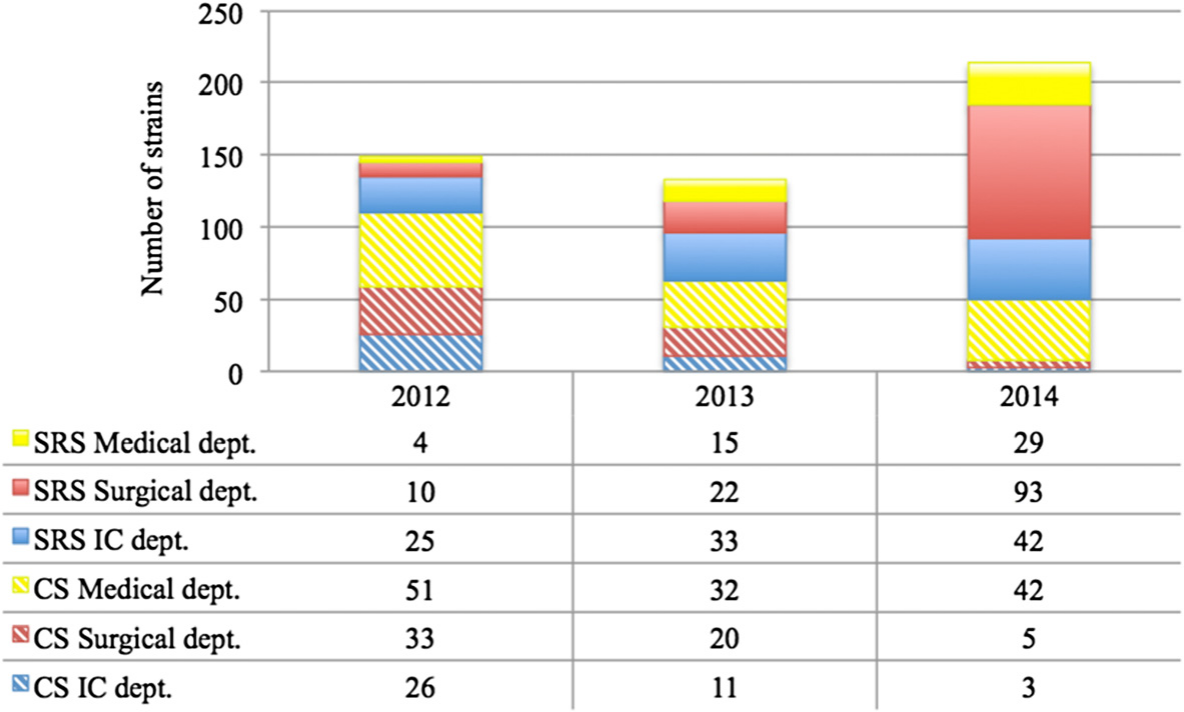
Carbapenemase producing *Klebsiella pneumoniae* strains isolated from surveillance rectal swabs and from clinical samples in 2012, 2013 and 2014. IC: intensive care. CS: clinical samples. SRS: surveillance rectal swabs

During the study period, 5,305 subjects were screened in the ICD, 7,560 were screened in the SD, and 2,239 patients were screened in the MD. The comparative analysis of the detection of CPKP by SRS versus CS revealed an increased rate of CPKP identified by SRS relative to CS (Fig. 2).

In the ICD, the percentage of cases identified by SRS was 49 % in 2012, 75 % in 2013 and 93.3 % in 2014 (the Chi-squared test for trend, *p* < 0.0001); a comparable figure was described in the SD, with the highest value (94.9 %) reported in 2014, and lower percentages in 2013 (52,4 %, *p* < 0.0001 respect to 2014) and in 2012 (23.2 %, *p* < 0.0001 respect to 2014 and *p* = 0.01 respect to 2013). The MD department was characterized by a sharp increase between 2012 (7.3 %) and both 2013 (31.9 %, *p* = 0.003) and 2014 (40.8 %, *p* = 0.0001).

In the ICD, this trend increased over time (from 1.5 % in 2012 to 1.8 % in 2013, and to 2.3 % in 2014). A statistically significant difference was demonstrated in the SD between 2012 and 2013 (1.9 % versus 0.8 %, *p* = 0.0357) and between 2013 and 2014 (0.8 % versus 2.2 % *p* < 0.0001); in the MD the increase from 2012 (0.8 %) to 2014 (2.6 %) was significant (*p* = 0.032) (Table 1).

**Table 1:** Percentage of positive SRS respect to the number of the patients involved in the surveillance in the Medical Department, Surgical Department and Intensive Care Department in 2012, 2013 and 2014

	Medical Department			Surgical Department			Intensive Care Department		
Year	Total SRS (n)	Patients (n)	Percentage of positive SRS	Total SRS (n)	Patients (n)	Percentage of positive SRS	Total SRS (n)	Patients (n)	Percentage of positive SRS
2012	1870	486	0.8 %	924	520	1.9 %	4255	1639	1.5 %
2013	2105	630	2.4 %	5516	2767	0.8 %	5225	1852	1.8 %
2014	3207	1123	2.6 %	9441	4272	2.2 %	5501	1815	2.3 %

*SRS* Surveillance rectal swab

Time to first detection of SRS was analyzed by evaluating three different figures.

In 2013, 37 % of the SRS were collected as first sample within 7 days from admission to the hospital, 45 % after a negative survey of one sample at least, 17 % after a minimum stay of 7 days with no surveillance data available at least. Of note, 19 % of the SRS cases were found positive after a negative survey of three samples at least. About clinical samples, these rates were 33 %, 38 % and 29 % respectively.

In 2014, 27 % of the SRS were collected as first sample within 7 days from admission, 60 % after a negative survey of one sample at least, 13 % after 7 days at least with no survey data. About clinical samples, these rates were 52 %, 31 % and 17 % respectively. It has to be underlined that even more SRS cases than 2013, 31 %, were found positive after a negative survey of three samples at least.

The MLST analysis revealed the circulation of CPKP strains previously described in other studies, including ST-258 and ST-512. The abundance of certain prevalent types may be partially because investigations were conducted on episodes of temporal or spatial clusters. Nevertheless, some new ST variants were demonstrated: strains 510, 527, 868, 1081, 1207, 1326 and 1733. Interestingly, in August 2011, a new single locus variant (SLV) of ST-512 (ST-745) was identified. Thereafter, the variant quickly spread throughout the hospital. ST-745 was identified in 24 of 74 strains analyzed in 2011, when 125 patients were demonstrated as infected or colonized by CPKP. MLST data obtained in 2012, 2013 and 2014 are presented in Table 2.

**Table 2:** Description of the ST- identified in carbapenemase producing *Klebsiella pneumoniae* divided by Department in 2012 (a), 2013 (b) and 2014 (c)

(a) 2012						
	ST-258 (n)	ST-512 (n)	ST-745^2^ (n)	ST-15 (n)	ST-101 (n)	ST-868^2^ (n)
MD	3	1	5	-	-	-
SD	9	10	6	-	-	1
ICD	33	5	15	2	2	-
Total ^1^	45	16	26	2	2	1
(b) 2013						
	ST-258 (n)	ST-512 (n)	ST-745^2^ (n)	ST-15 (n)	ST-307 (n)	Other ST (n)
MD	10	5	1	-	-	
SD	10	15	5	-	3	392 (1 pt)^4^
ICD	16	21	4	3	2	437 (2 pts)
						1207^4^ (1 pt)
						1326^4^ (1 pt)
Total ^3^	36	41	10	3	5	5
(c) 2014						
	ST-258 (n)	ST-512 (n)	ST-745^2^ (n)	ST-307 (n)	ST-554 (n)	Other ST (n)
MD	1	3	-	-	1	
SD	7	15	1	-	-	395 (1 pt)
ICD	1	11	-	1	2	1199 (1 pt)
						1543 (1 pt)
Total^5^	9	29	1	1	3	3

^1^ MLST available for 92/149 isolates

^2^ single locus variant of ST-512

^3^ MLST available for 100/133 isolates

^4^ new allelic profile

^5^ MLST available for 46/214 isolates

*MD* Medical Department, *SD* Surgical Department, *ICD* Intensive Care Department, *pt* patient, *pts* patients

### Colistin-resistant strains

**Fig. 3:**
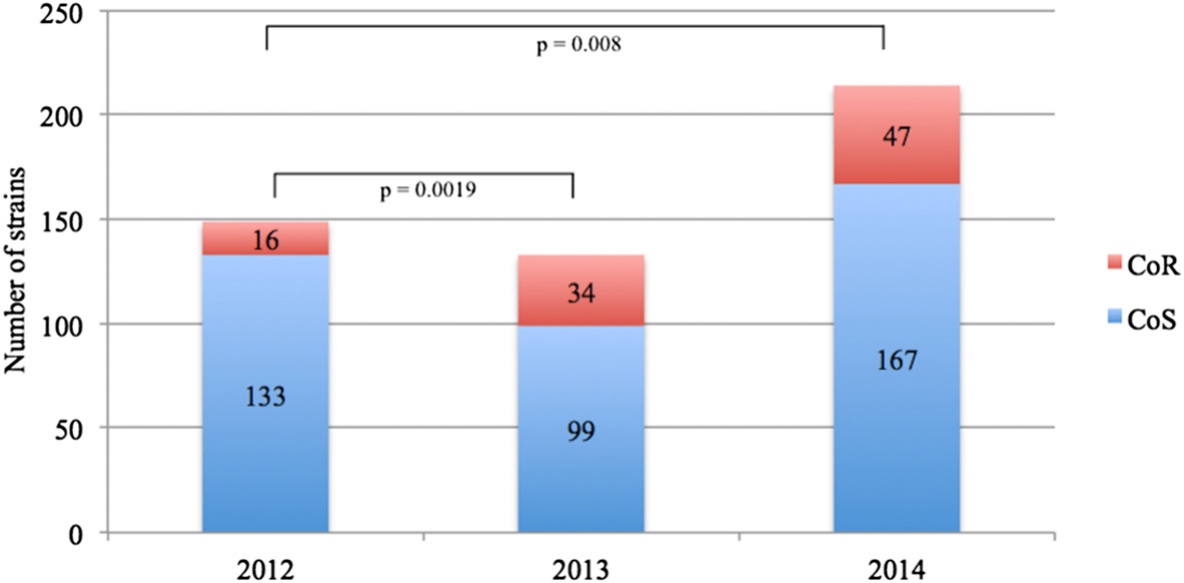
Number of colistin resistant strains and colistin susceptible strains identified in 2012, 2013 and 2014. CoR: colistin resistant. CoS: colistin susceptible

At the first detection in our survey, we collected a total of 399 CoS strains and 97 CoR strains (Fig. 3). The percentage of CoR cases (both SRS and CS) relative to all CPKP cases was 10.7 % in 2012: a statistically significant increase respect to 2012 occurred in 2013 (25.6 %) and in 2014 (22 %).

**Fig. 4:**
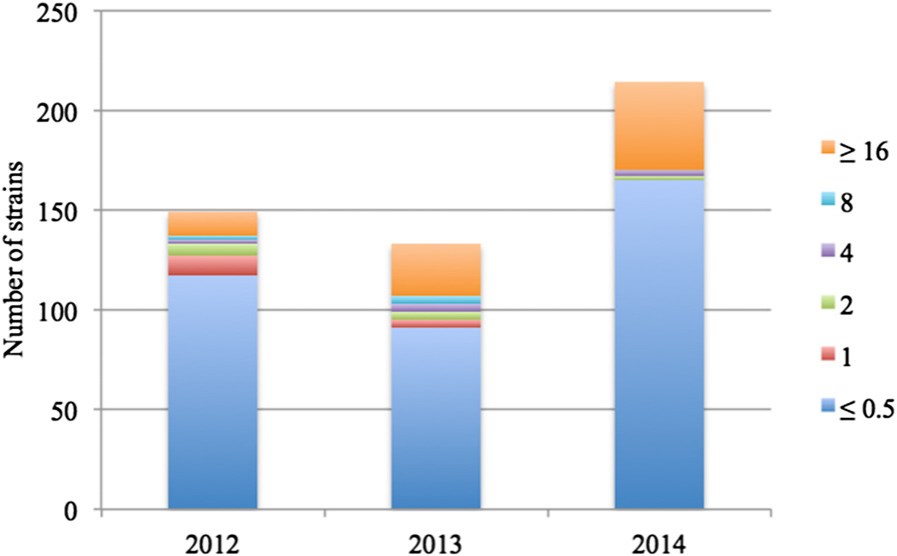
Colistin MIC values (mg/L) performed with Vitek® 2 system in 2012, 2013 and 2014. MIC: minimum inhibitory concentration

**Fig. 5:**
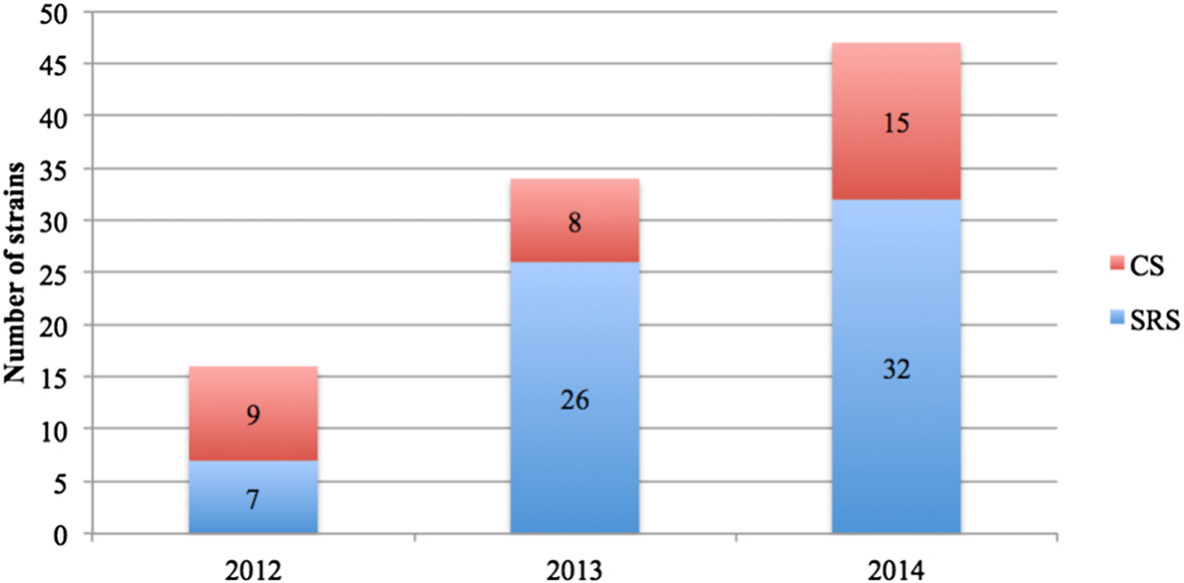
Colistin resistant strains at the first detection in 2012, 2013 and 2014. CS: clinical samples. SRS: surveillance rectal samples

The following overall distribution of MIC values was observed after screening with Vitek: 372 strains exhibited an MIC value ≤ 0.5 mg/L, 14 strains were MIC = 1 mg/L, 12 strains were MIC = 2 mg/L, 9 strains were MIC = 4 mg/L, 6 strains were MIC = 8 mg/L, and 83 strains were MIC ≥ 16 mg/L. A detailed description (by year) is provided in Fig. 4. Among the strains classified as CoR at the first detection, 65 and 32 were identified by SRS and CS, respectively (Fig. 5). No differences were observed in the CS and SRS MIC values. With respect to the clinical samples, 12 of the 32 strains were isolated from urine cultures, 7 strains were detected in bronchial aspirates, 6 strains from skin swabs, 2 strains from blood cultures, 2 strains in drainage fluid, 2 strains from vaginal swabs and 1 strain from a pharyngeal swab (Table 3). Among the 65 SRS that were found to be CoR, 38 % were collected from the patient’s first SR. The increase proportion of CoR strains isolated at the first detection from SRS in 2013 compared with 2012 was statistically significant (*p* = 0.02) Moreover, we observed a significantly higher frequency of CoR strains detected from SRS when we compare them respect to the number of all available CPKP cases (*p* = 0.0002 in 2012 versus 2013, *p* = 0.0034 in 2012 versus 2014).

**Table 3:** Description of the first detection of a colistin resistant strain in clinical samples in 2012, 2013 and 2014

Year	Clinical Samples						
	Urine (n)	Bronchial aspirates (n)	Skin swab (n)	Blood (n)	Drainage fluid (n)	Vaginal swab (n)	Pharyngeal swab (n)
2012	1	4	1	1	1	0	1
2013	4	1	2	0	1	0	0
2014	7	2	3	1	0	2	0

### Evolution of susceptibility to colistin

Although not from the same material (FU), a strain that was later isolated in the same patient was also detected in 210/399 patients with a CoS stains at first detection, allowing us to perform a longitudinal analysis. After previously identifying a CoS strain, we identified a CoR strain in 50/210 patients (23.8 %) (Table 4). We were able to perform MLST for both the CoS and CoR strains in 17 of 50 patients (Table 5). All pairs of isolates were obtained in 2014, with the exception of the strain from patient 14 that was detected in 2013. The same ST was detected in both the CoS and CoR strains in 13/18 strains. In 11/13 cases, the CoS strains was also isolated on CS. In one case, another pathogen (*Enterobacter aerogenes,* susceptible only to colistin) was isolated from a CS. None of these patients were discharged within the time interval between the two isolates.

**Table 4:** Longitudinal analysis of colistin susceptibility evolution in patients with a colistin susceptible isolate at the first detection in 2012, 2013 and 2014

Year	CoS at first detection (n)	Patients with FU available (n)	CoS at FU (n)	CoR at FU (n,%)
2012	133	71	59	12 (16.9 %)
2013	99	57	39	18 (31.6 %)
2014	167	82	62	20 (24.4 %)

*CoS* Colistin susceptible, *CoR* Colistin resistant, *FU* follow-up

**Table 5:** MLST analysis on pairs of CoS and CoR strains obtained from the same patient. Time to switch is intended as the time-interval between the first CoS strain isolated from a clinical sample and the first CoR strain isolated from the same patient

	Strains with Stable ST					
Patient	ST of the first CoS strain isolated	Sample type	Sample type of first clinical CoS	ST of CoR strain isolated	Sample type	Time to switch from CoR to CoS (days)
1	554	SRS	Arterial catheter	-	bronchial aspirate	36
2	512	SRS	skin swab	-	urine	26
3	307	SRS	skin swab	-	skin swab	42
4	512	SRS	skin swab	-	SRS	123
5	512	SRS	drainage fluid	-	drainage fluid	28
6	512	urine	urine	-	urine	17
7	512	SRS	bronchial aspirate	-	SRS	74
8	1543	SRS	-	-	SRS	50
9	512	SRS	bronchial aspirate	-	bronchial aspirate	31
10	512	SRS	bronchial aspirate	-	bronchial aspirate	6
11	554	SRS	-	-	bronchial aspirate	na
12	512	SRS	bronchial aspirate	-	blood	24
13	258	SRS	urine	-	urine	8
						Median
						29.5 days
	Switched ST					
Patient	ST of the first CoS strain isolated	Sample type	Sample type of first clinical CoS	ST of CoR strain isolated	Sample type	Time to switch from CoR to CoS (days)
14	15	SRS	urine	512	SRS	167
15	554	urine	urine	512	SRS	67
16	258^1^	SRS	skin swab	512	SRS	34
17	554	SRS	arterial catheter	1733 (554 SLV)	drainage fluid	54
						Median:
						60.5 days

^1^ simultaneous isolation of a ST258 CoS strains and a ST512 CoR strain from the same sample at the first detection”

*CoR* colistin resistant, *CoS* colistin susceptible, *MLST* Multilocus sequence typing, *na* not applicable, *SLV* single locus variant, *SRS* surveillance rectal swab, *ST* sequence typing

Four of 18 strains exhibited a different ST between the CoS and CoR strains. Two patients were discharged during the FU, and 2 other patients were continuously hospitalized. Note that in one of these four cases, the CoR strain (ST1733) was a single locus variant of the CoS strain (ST554). Patient 16 reported a simultaneous isolation of a ST258 CoS strains and of a ST512 CoR strain from the same sample at the first detection.

All cases were tested to identify the KPC type; in 15 couples both isolates were KPC-3 and in one couple both strains were KPC-2, suggesting a persisting infection with the same strain. In one case we found a KPC-2 at baseline and a KPC-3 at follow-up: this finding made difficult a definite interpretation.

### Isolation of CPKP from blood

Seventy-eight CPKP strains were isolated from the blood of 75 patients as a first or subsequent detection: 32 in 2012 (5 CoR), 17 in 2013 (1 CoR) and 29 (6 CoR) in 2014. The crude mortality rate among patients with CPKP isolated from the blood was 35/75 (46.7 %). In cases of poor outcomes, the median time to death was 10 days, whereas the median time to discharge was 28 days.

## Discussion

In this single-center observational analysis, we described the changes observed over time (3 years) in the frequency of CPKP detection in clinical samples and on SRS. Furthermore, we analyzed the trend in CPKP colistin susceptibility and the evolution of colistin-sensitive to colistin-resistant strains over time.

Two strengths of this work are the updated reports (December 2014) and the study design. The screening program included 15,104 patients in three different clinical settings at the same hospital (ICD, SD and MD), and comparable infection control strategies were applied in each setting.

In 2014, we observed a statistically significant increase in the percentage of patients in whom CPKP was detected by SRS (2.3 %), compared with 2012 (1.5 %) and 2013 (1.3 %). The frequency reported was lower than that observed by Gagliotti et al. [20], who reported a 3.9 % rate in a cohort of 1,687 patients from August to December 2011; however, the characteristics of the screened subjects are only somewhat comparable. Gagliotti et al. included only those subjects who were transferred from other institutions (both hospitals and long-term care facilities) or who were hospitalized in the previous two months or recovered in ICDs or post-acute wards, whereas we applied no exclusion criteria to the surgical subjects. Moreover, we expanded the cohorts of the involved patients over time according to their clinical needs and implemented effective strategies to prevent infection. The effect of this evolving pattern of surveillance was demonstrated by the significant trend in increased proportions of CPKP first isolated from SRS relative to those detected in clinical samples from the three departments.

The increased surveillance schedule in the MD was matched by a significant increase in positivity from 2012 to 2014. However, the important increase in the number cases under surveillance in the SD (520 subjects in 2012, 2,767 in 2013 and 4,272 in 2014) did not result in a decrease in the percentage of positive cases (1.9 % in 2012 and 2.2 % in 2014). This finding underlines the major role of an early identification of the colonized or infected patients and of a programmed recall of the recognized measures to prevent transmission in all the clinical settings. ICD stay is a well-known independent risk factor for colonization with carbapenem-resistant *K. pneumoniae* [21]. Nevertheless, if we evaluate the percentage of patients identified as carriers in 2014, comparable data were reported in three different departments (2.3 % in the ICD, 2.2 % in the SD and 2.6 % in the MD).

About the modality of acquisition of KPC, we cannot exclude a previous positivity at entry in about 15 % of SRS positive patients and in about 28 % of the patients with a positive CS. Furthermore, we have to take into account the low susceptibility of a survey by rectal swab, possibly due to an intermittent positivity, to a low bacterial load or to an incorrect sampling. Nevertheless about at least 26 % of cases with a detection in SRS were found positive after a previous prolonged negative survey and with a possible acquisition of KPC after the admission to the hospital.

These data justify and support the survey at entry and the continuation of the surveillance throughout the hospitalization, in a setting at high prevalence as the one we described, for a better management of the colonized subject and to limit the spread to other patients. About the use of MLST in our cases, although the coverage of this analysis was 61.7 % in 2012 and 75.2 % in 2013, we were allowed to trace and confirm some clusters of infection, which was useful for legal reasons, and we could detect some new ST-variants. One of these suddenly spread over the hospital, and molecular typing allowed us to analyze and reform some procedures and to sensitibilize health care workers to the problem. Typing also allowed us to understand the multifaceted nature of our KPC population. Finally, we were able to identify and correctly interpret at least one case of co-infection with two different ST and three cases of superinfection with a CoR strain.

The emergence of colistin resistance may be related to drug-selection pressure, but horizontal transmission is a recognized cause of acquisition, and gene transfer factors (i.e., transposons and integrons) may also be involved [22–24].

In 2014, the CoR detection rate (including both SRS and CS) was 22 % among all CPKP cases. Monaco et al. [8] reported a higher value (43 %) in a multicenter Italian survey, but most cases were obtained from clinical isolates. Thirty-two of the 47 resistant strains we identified in 2014 (68.1 %) were identified by SRS, indicating a potential clinical risk for the colonized subject. A later infection was described in 9 % of patients admitted to an Israeli tertiary care hospital, with surveillance rectal cultures that were positive for carbapenem-resistant *Klebsiella pneumoniae* after a median of 20 days [25]. Approximately half of the 15 clinical samples (46.7 %) in which a CoR strain was detected were urine samples, which included both asymptomatic bacteriuria and clinical urinary tract infections. In the study by Shilo et al., patients who were diagnosed with CRKP bacteriuria at a general hospital exhibited a high mortality rate (29 %), perhaps because this positivity indicated worse prognosis [26]. When the CRKP is colistin resistant, the overall mortality rate is higher (40.6 %), and the presence of infection is an independent negative prognostic factor [27].

The detection may be a consequence of previous hospitalization, likely because of prior antibiotic treatment. Furthermore, possible patient-to-patient transmission is possible even outside the healthcare setting if the carrier status is unknown due to the long-term persistence (up to several months) of the colonization by carbapenemase-producing enterobacteria [28, 29]. In addition, possible horizontal transmission of colistin resistance from farm animals to humans in Asia has been suggested by Olaitana et al. [30], and the potential transfer of resistance genes between species must be considered due to the extensive use of antibiotics in veterinary medicine [31].

We identified a CoR strain in approximately one quarter of the patients who exhibited a CoS strain at the first diagnosis; a follow-up control sample is available. Colistin has emerged as an effective treatment option for multidrug resistant infections; however, drug-selection pressure pressure probably led to the development of resistance [32]. The total duration of therapy plays no role [33], but prolonged monotherapy or suboptimal dosing can contribute to the development of resistance [34]. Furthermore, acquired colistin resistance might persist in the carrier and in the environment. Bogdanovich et al. demonstrated that colistin-resistant CPKP strains persist in cultures for 10 days in Luria-Bertani broth with no colistin supplementation, which confirms the need for repeated SRS checks during hospital stays [22]. In select cases, molecular screening should be performed together with culture testing. In our study, 4 of the 17 patients with a CoS first isolate and follow-up CoR isolate exhibited different ST; two of these patients were never discharged from the hospital. We can assume that a superinfection developed in these subjects rather than an evolution of the strain caused by drug-selection pressure. In all but one case the two strains the same KPC variant was detected.

A limitation of this study is the lack of colistin treatment data. However, the aim of this report was to describe the phenomenon of CPKP detection in clinical samples and SRS in a tertiary care hospital as well as to report the trend of colistin resistance in three different care settings and in patients over time.

## Conclusions

We observed a progressive increase in the detection of CPKP strains in the screening samples as well as a greater number of CoR strains at the first identification, thus demonstrating higher selection pressure for colistin resistance. A colistin-resistant strain was detected in approximately one-quarter of the patients with a previous CoS isolation, and according to the available longitudinal data, a relevant and increasing proportion of CoR strains was found in the surveillance rectal swabs; this finding supports the clinical relevance of surveillance rectal swabs.

## Abbreviations

CoR: Colistin resistant
CoS: Colistin susceptible
CPKP: Carbapenemase producing *Klebsiella pneumoniae*
CS: Clinical samples
ESBL: Extended spectrum beta-lactamase
ICD: Intensive Care Department
MD: Medical Department
MIC: Minimum inhibitory concentration
MLST: Multilocus sequence typing
NDM: New Delhi metallo-β-lactamase types
SD: Surgery Department
SLV: Single locus variant
SRS: Surveillance rectal swabs
VIM: Verona integron-encoded metallo-β-lactamase
